# Welfare Indicators in Tilapia: An Epidemiological Approach

**DOI:** 10.3389/fvets.2022.882567

**Published:** 2022-06-27

**Authors:** Luis Flores-García, Juan C. Camargo-Castellanos, Cristina Pascual-Jímenez, Pablo Almazán-Rueda, Jorge Francisco Monroy-López, Pedro J. Albertos-Alpuche, Rosario Martínez-Yáñez

**Affiliations:** ^1^Biosciences Doctoral Program, Universidad de Guanajuato, Irapuato, Mexico; ^2^Unidad Multidisciplinaria de Docencia e Investigación, Facultad de Ciencias, Universidad Nacional Autónoma de México, Hunucmá, Mexico; ^3^Centro de Investigación en Alimentación y Desarrollo, Mazatlán, Mexico; ^4^Departamento de Medicina Preventiva y Salud Pública, Facultad de Medicina Veterinaria y Zootecnia, Universidad Nacional Autónoma de México, Mexico City, Mexico; ^5^Aquaculture Laboratory, Universidad de Guanajuato, Irapuato, Mexico

**Keywords:** welfare indicator, *Oreochromis niloticus*, populational risk, incidence, epidemiological approach

## Abstract

Interest and concern about rearing methods and their impact on animal welfare have increased. Production evaluation is population-based, and animal welfare analysis should be similar. In fish, the most common welfare indicators are gill state, fin damage, and body condition. The objective of this study was to evaluate the feeding rate effect on the welfare indicators of *Oreochromis niloticus* using an epidemiological approach. Five growth stages (from 1.2 to 360 g) were studied using four feeding rates as treatments: underfeeding (80%), recommended feeding (100%), and two levels of overfeeding (120% and 140%). The evaluated welfare indicators include the presence of lesions in different body areas and fins, the decrease in body condition index, and their impact on biomass production. Incidence and relative risk were determined for each indicator. Statistically significant associations were found in the indicators of mortality, weight, body condition (K), and presence of evident damage in the caudal and anal fin in all stages. The results showed that the feed rate directly affects the welfare indicators and production. Mortality, weight reduction, K reduction, and caudal and anal fin damage incidence showed to be relevant indicators in all *O. niloticus* growing stages. As a result of this study, the epidemiological approach seems to be a valuable tool for production. A risk traffic light method is a proposal that could have great potential, with the suggested limits for WI's concerning the individuals present in the culture pond, allowing progressive evaluation and decision-making to correct risky situations.

## Introduction

The main goal of animal production is to obtain protein for human consumption. As in terrestrial animals, in fish, the diet must consider various factors such as age and growth stage, since incorrect management will cause individuals to have few opportunities to develop correctly, regardless of the species (1). The demand for food is growing as a response to the increase in the human population, as it is estimated that by the year 2050 it will reach around nine billion people (2); this implies an enormous challenge for the primary production sectors, which are increasingly under pressure to satisfy this need. Consumption of aquatic animal protein has increased 15% in the last 10 years; in 2018, aquaculture contributed 82.1 million tons of biomass for human consumption, 54.3 million of these came from fish, tilapia (*Oreochromis spp*.) being one of the most important species for freshwater aquaculture (2). On the other hand, the decrease in water availability means that animal production systems must be substantially modified, to make effective and efficient use of water resources and carry out sustainable production. Recirculating aquaculture systems (RAS) are frequent in semi-intensive and intensive aquaculture, based on a cyclical water movement. They consist of taking the water from a pond, passing it through filters, and returning it to the pond already clean. Such systems mean an enormous advantage in saving water, especially in regions where it is scarce. Physicochemical parameters do not have significant variations and prevent diseases from spreading to the entire production unit (3). This technique has been proposed as a sustainable alternative for the efficient use of water and the environmental impact reduction associated with aquaculture.

The public interest and concern about the raising methods and their impact on the welfare of production animals has increased globally, and fish production is no exception. Welfare is defined as the dynamic state of an individual concerning the biological mechanisms used to adapt positively and successfully to changes in the environment, involving health (4), comfort (5), and the emotional state of the animals (4–6). The farmer must be responsible to provide calmness, comfort, protection, and safety to the farmed animals, during their breeding, maintenance, production, transport, and slaughter (7). Like terrestrial production species, aquatic animals require specific management and growth conditions according to the species and life stage. To provide adequate welfare levels, fish farmers must observe, measure, and control various variables such as water quality, population density, and feeding practices (8). According to Mellor et al. (9), the five domains model for the evaluation of animal welfare describes the sum and interaction of the variables related to survival (1: nutrition, 2: environment, and 3: health) and the situational variables (4: behavior), it directly infers on the mental state of the individuals (domain no. 5), which, in turn, allows qualifying the welfare state of the animals at a given moment.

The behavior and welfare of fish have been the subject of debate for years. In 2002, the United Kingdom implemented laws on the management of salmon farm production to improve the living conditions of the animals, resulting in better-quality products (10). Norway in 2005 started regulating aquaculture production with guidelines like those implemented by the UK (11). Recent research has shown that fish welfare is strongly related to fish physiology, which impacts production and considers animal welfare as a key element for the expression of the full genetic production potential of farmed fish (8, 12, 13). Fish possess homeostatic mechanisms that allow individuals to adapt to their environment, through physiological changes both internal and external (14). To know the welfare state in a fish, welfare indicators (WI's) can be used. These can be determined directly on the animals, such as fins condition body deformations, or indirectly, which are mainly environmental conditions. Once WIs are used as standard on laboratories or farms, they become laboratory welfare indicators or operational welfare indicators (10). Most studies coincide that the best and most widely used WIs are individual-based (or direct) welfare indicators, which can be determined both at the farm and laboratory level (10). The most common of these indicators are operculum beating rate, reflex behavior, gill status, condition factor, fin damage, and body integrity (13, 14). Group based welfare indicators most used are the mortality rate (15), swimming behavior (16), appetite (17), growth rate (18), presence of diseases (10), presence of scales or blood in the water (10), the state of the fins, the integrity of the body, and the body condition (19). Fins are anatomical structures that help in the mobility of the fish, therefore, the integrity of these – mainly the dorsal, lateral, and caudal fins – are indicators of health and welfare (10, 16, 19). In fish, the condition factor (K) is a well-accepted tool for assessing the nutritional status (18), overall quality (20), and feeding management (16, 18). Body condition is variable throughout the lives of fish; thus, it is difficult to define exact values that are indicative of reduced welfare. However, <0.9 is usually indicative of emaciation (21). Feed management in aquaculture farms requires a significant amount of resources, and labor, consequently, represents an important production cost. The feeding rate (amount of feed supplied) is determined in relation to the biomass contained in a fish tank, cage, or pond (16). In the case of tilapia production, as well as in other farmed fish, the feeding is given using standardized feeding tables according to the growth stage (22). Likewise, the companies that manufacture balanced fish feed issue tables of feeding programs, where the suggested handling rate is indicated according to the weight of the organisms and the product. Feeding practices that affect fish welfare also include feeding schedules (23, 24). During production, erroneous management such as underfeeding and overfeeding can occur with negative effects on the welfare of fish, either due to lack of nutrients (25) or deterioration in water quality (25–27).

Epidemiology is the study of disease in populations and of factors that determine its occurrence, the keyword being populations (28). Veterinary epidemiology additionally includes research and assessment of other health-related events, notably productivity (29). All this research involves observing animal populations and making inferences from the observations (29). Epidemiological tools are useful when studying the general status of a certain group, establishing diagnostic criteria to carry out evaluations that allow the prevention, detection, correction, and control of problems, particularly health problems. Epidemiological indicators are calculations used to determine the exposure of a population to a disease or any damage, such as body areas with descaling, hemorrhages, or broken fins (lesion), that is, the probability of the presence of a specific event in a defined time. A cohort study (prospective) is based on the evaluation of the occurrence of an event (in terms of presence/absence) as a result of the follow-up over time of a group, as a consequence of having been exposed or not (comparison groups) to a certain exposure (risk factor) (28, 29). The analysis of the probability of an event occurrence, using epidemiological indicators such as *odds ratio*, to identify risk factors for the presence of bodily injuries and their impact on welfare, has recently been reported in terrestrial animals (30). The incidence (I) represents the number of cases (events) that appear in a population and in each period of time (28). The relative risk (RR) is a measure of the relationship existing between the probability that an event occurs in the exposed group with the same risk factor. The RR is calculated from the cases (events) observed in the group of animals exposed to the risk factor in relation to the cases (events) observed in the group of animals not exposed to the risk factor. It is essential to calculate the corresponding confidence intervals (CIs), which allows for giving greater statistical weight to the calculated RR value. The CI values allow a correct interpretation of the RR result obtained because they approximate to the real value in the population under study since this is inaccessible but it is located inbetween the CI range, with a degree of uncertainty that we can determine (95%) (29, 31). When the RR is equal to or >1.0, then there is a negative effect on the risk factor for the incidence of the event; when it is <1.0, there is no such negative effect on the population (28, 31).

Aquaculture production is generally evaluated considering the population, losing the richness of individual values (8, 13, 17, 32). Therefore, the advantages of analyzing WIs with epidemiological statistical tools could allow measuring the state of a determined group of fish, advancing the knowledge of the animal welfare and the application of WIs in farms or laboratories. Mortality, body condition, damage to the eyes, mouth, opercula, skin, degree of scaling bleeding lesions, and damage such as tears, fraying or bleeding that affects the integrity of the dorsal, lateral, anal, and caudal fins have been part of proposals for the evaluation of welfare indicators in fish (12–14), which have been put into operational practice by applying in *Salmo salar* (10, 33), *Perca fluviatilis* (34), and *Oreochromis niloticus* (35), along with other indicators such as water quality and production rates. Some studies report WIs in the proportion of the damage in an evaluated population (10, 33–35), but in these researches, an analysis is not carried out to determine if the degree of damage registered is considered a situation of population risk, as it would be done in analysis with epidemiological statistical tools. Therefore, the objective of this study was to evaluate the effect of the feeding rate on the welfare indicators of tilapia (*Oreochromis niloticus*) cultivated in recirculating aquaculture systems, using an epidemiological approach.

## Materials and Methods

### Location

The study was carried out at the facilities of the Aquaculture Laboratory of the Veterinary and Zootechnical Department of the Life Sciences Division, Campus Irapuato-Salamanca of the University of Guanajuato, located according to Geo Locator (2021) at 20 ° 44′34.65″N and 101 ° 19′51.78″W, at 1,745 meters above sea level. The study was carried out from April to November 2019.

### Experimental Systems

Twelve individual and independent experimental conventional aquaculture recirculation systems (RAS) were used (*n* = 3 per treatment), which were located inside a greenhouse and were built based on the modified design of Timmons and Ebeling (3). At the beginning of each experiment, a total water change, and deep cleaning of the components were carried out. Each system consisted of a 1.5 m^3^ fish tank with an integrated filter of four elements with a capacity of 0.2 m^3^ each (1 settler-clarifier, 2 physical particle separations with filter material inside, and 1 biological with biospheres). Water lines were 2” hydraulic PVC pipes. The internal movement of water was carried out with a submersible pump (RESUN Model SP3800, Q = 2,000 L/h) placed inside the biological filter. To maintain a constant and suitable temperature for the species, each tank was covered with a dome made of ¼” plastic and PVC, with a small opening. Air was injected at a rate of 40 L/min to each tank and biological filter, using aerating stones connected to a general distribution line to all systems and to a compressor (RESUN GF-750). The RAS were filled to their maximum capacity the same day with water from a single well. The movement and oxygenation of the water began, after 24 hours, adding lyophilized bacteria (AZOO-NitriPro, Nitrosomonas, and Nitrobacter) at a rate of 3 g per 250 L of water, following the protocol described by Espinoza-Moya et al. (36).

### Fish and Food

The project was evaluated and approved by the Institutional Committee of Bioethics in Research of the University of Guanajuato (code: CIBIUG-A59-2020). The specimens of *O. niloticus* (males obtained by sexual reversion) were purchased from a commercial farm located in Chupícuaro, Guanajuato. The fish were transported with the farm's water, inside plastic bags with oxygen injection, and placed in a reception tank (quarantine) for their acclimatization (14 days). Five experiments were carried out, in 6 months. Each growth stage of the tilapia (5 stages in total) was considered a separate experiment. The growth stages (experiments) according to the initial weight of the fish were in the following ranges: fingerlings (1.2–1.3 g), juveniles (9.0–9.5 g), on-growing 1 (34–37 g), on-growing 2 (65–70 g) and on-growing 3 (130–150 g). Fish size and density were considered for fish management (37). The experimental design: initial values of the number of fish per pond per experiment (n) and their characteristics (average initial values ± SD of weight, length, and K), initial biomass per pond (average initial weight ± SD and % CV), feeding rates treatments (%), feed characteristics and management (species-specific commercial balance), and duration (days) of each experiment can be seen in Table 1.

**Table 1:** Experimental design: initial values of the number of fish per pond, weight, length, and K.

	**Experiments**				
	**Fingerlings**	**Juvenile**	**On-growing 1**	**On-growing 2**	**On-growing 3**
**Fish**					
n per pond	180	100	100	100	60
Weight (g)^a^	1.26 ± 0.03	9.26 ± 0.19	35.13 ± 0.82	66.89 ± 2.07	144.84 ± 10.14
Length (cm)^a^	3.86 ± 0.31	7.84 ± 0.07	12.00 ± 0.21	14.99 ± 0.22	19.14 ± 0.49
K^1^	2.32 ± 0.53	1.92 ± 0.02	2.04 ± 0.07	1.99 ± 0.04	2.07 ± 0.03
**Pond**					
Biomass weight (k)^b^	0.22 ± 0.006 2.91%	0.92 ± 0.02 2.07%	3.51 ± 0.08 2.32%	6.68 ± 0.20 3.10%	8.78 ± 0.55 6.32%
**Feeding rates treatments, %**					
Underfeeding	6.4	4.0	3.2	3.2	2.4
Control^*^	8.0	5.0	4.0	4.0	3.0
Overfeeding A	9.6	6.0	4.8	4.8	3.6
Overfeeding B	11.2	7.0	5.6	5.6	4.2
**Feed**					
Characteristics	DM: 95.04 CP: 49.18 GE: 4.51 PS: <0.35	DM: 95.26 PC: 47.66 GE: 4.84 PS: 1.5	DM: 94.21 PC: 44.38 GE: 4.36 PS: 2.4	DM: 94.04 PC: 40.27 GE: 4.14 PS: 3.5	DM: 92.47 PC: 36.51 GE: 3.99 PS: 4.8
Servings a day	6	5	5	5	4
**Duration**					
Days	24	27	21	29	60

a *Values ± SD*;

b *Values ± SD, % CV. DM, Dry Matter (%); PC, Protein Crude (%); GE, Gross energy (Kcal/g); PS, Particle Size (mm); balanced specific for the species*.

* *Recommended (22), feeding rate in relation to the pond biomass. Replicates per treatment = 3 ponds*.

*Initial biomass per pond, feeding rates treatments (%), feed characteristics and management, and duration (days) of each experiment*.

Data were obtained from a total of 6,480 tilapias. At the beginning and end of each growth stage total fish length was recorded using an ichthyometer (Pentair Aquatic Ecosystems Inc.). Wet live weight (g) was recorded using a digital scale (RHINO, model BAPRE-3). At the end of each experiment, the same measurements were recorded and the same equipment was used to obtain all animal-related data. Due to the high number of specimens used per treatment, metabolism was decreased (lethargy and tranquillization) using cold water to weigh, measure, and check the animals externally. For both initial and end experiment data collection we followed the same pathway. First, we proceeded individually to reduce the individual's metabolism with cold water, making a sudden change in the maintenance temperature to 5°C (where swimming pattern change is visible), this being a recommended method to calm and immobilize fish (38, 39). The data collection and photographs did not exceed 90 seconds for each animal. Immediately after this, tilapias were put in a container with air injection for their recovery, and awakened fish that showed normal respiration and swimming were transported to a community 10,000 L RAS pond (under similar management conditions to the control treatment). It should be noted that the same set of animals did not participate in successive experiments. Four feeding rates were used as treatments: underfeeding (Ufe, 80%), recommended feeding (22) (Control, 100%), and two levels of overfeeding (OfA: 120% and OfB: 140%). Balanced feed samples were taken to determine crude protein (CP) (AOAC) and gross energy (GE) by combustion (IKA Calorimeter System C 2000 Basic). To adjust the amount of feed to be supplied, a sample was taken each week from the animals which were weighed, mortality was also considered for each experimental system.

### Production Variables and Water Quality

For each pond, total biomass production (g) was determined as the final harvested weight minus the initial weight. Water quality was measured, at 9:00 am every other day, variables were temperature (°C), dissolved oxygen (mg L^−1^), electrical conductivity (mS/cm^3^), ${\text{NH}}_{4}^{+}$ (mg L^−1^), and pH, using a multiparameter recorder (YSI Mod. Professional Plus, Cable Quatro). Measurements were carried out directly in the tank in the middle of the water column.

### Welfare Indicators (WIs) and Epidemiological Approach

A thorough visual inspection was performed to determine the presence or absence of visible damage to the eyes, mouth, opercula, skin, descaling, bleeding, and dorsal, lateral, anal, and caudal fin damage measured as tears, fraying, or bleeding (16) (without damage: 0, damaged: 1). Mortality (alive: 0, death: 1), weight reduction and body condition index (Fulton's K) were calculated (higher: 0, lower: 1) (Table 2).


$$\begin{array}{l} Mortality=no.\;of\;harvested\;fish-no.\;of\;fish\;stocked \end{array}$$

To determine the number of fish with an increase or decrease in weight or K, at the end of each experiment, per pond per treatment, the average value of control treatment per fish per pond was taken, as follows:


$$\begin{array}{l} Final\;weight\;per\;fish-\overset{-}{x}\;weight\;control\;treatment \end{array}$$

where the positive values are taken as an increase (0) and the negative values as a decrease (1) in weight per fish (Table 2).


$$\begin{array}{l} Fultons\;K=\left(W/L^{3}\right) \end{array}$$

where, W is the individual wet weight (g), and L is the total length (cm).

**Table 2:** Description of the welfare indicator (index description) evaluated.

**INCIDENCE**	(nO/nTx)*100	
**Welfare Indicator** **(Index description)**	**Formula application**	**Visual**
**Mortality incidence:** Number of dead fish	**nO:** no. of individuals from the treatment who died **nTx:** no. of individuals per treatment	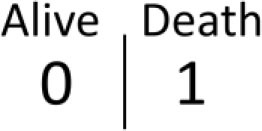
**Weight reduction incidence:** Number of fish weighing less than the average of the treatments Control	**nO:** no. of individuals in the treatment who presented lower body weight than the average of the control **nTx:** no. of individuals per treatment	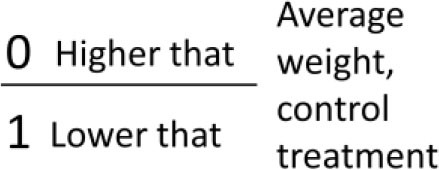
		**Note:** On farms it is recommended to use control data obtained in successful production cycles
**K reduction incidence:** Number of fish with lower K than the initial one per pond	**nO:** No. of individuals in the treatment who presented lower K than the initial value **nTx:** no. of individuals per treatment	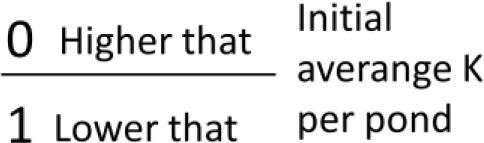
**Caudal/Anal fin damage incidence:** Number of fish with presence of damage to caudal and anal fins	**nO:** No. of treatment individuals who presented damage to the caudal and / or anal fin **nTx:** no. f individuals per treatment	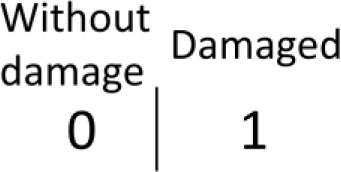
Caudal fin without damage	Damaged caudal fin (numbers from ichthyometer)	Damaged caudal fin
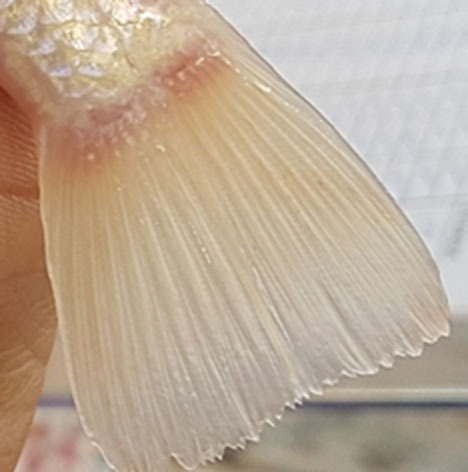	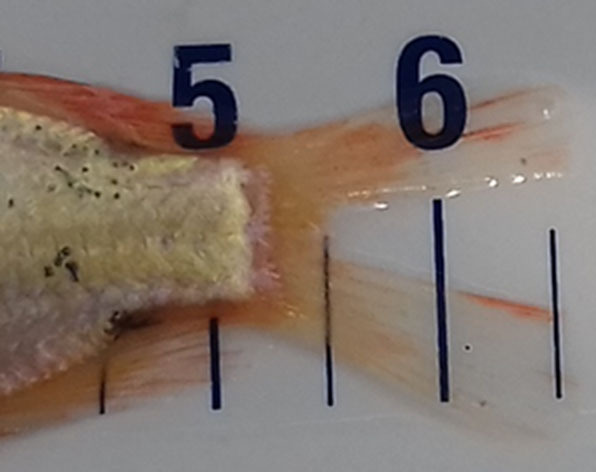	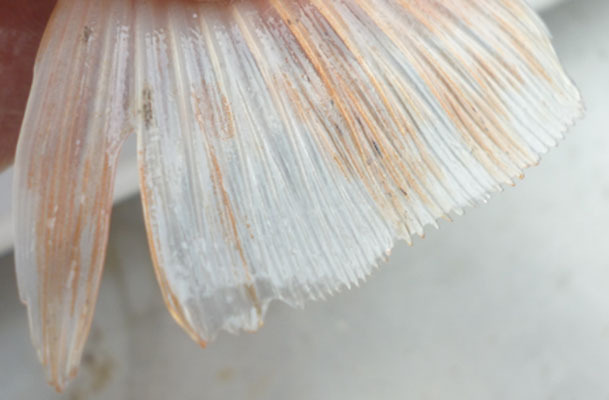
Anal fin without damage	Hemorrhagic anal fin	Damaged anal fin
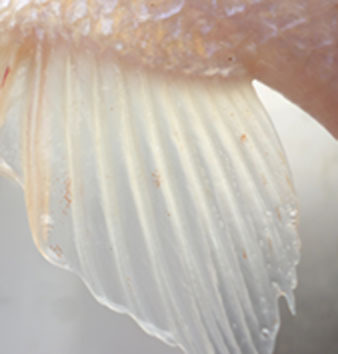	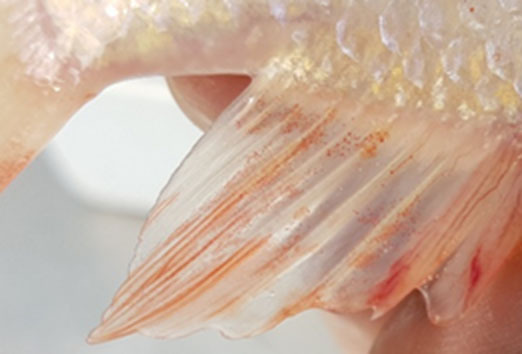	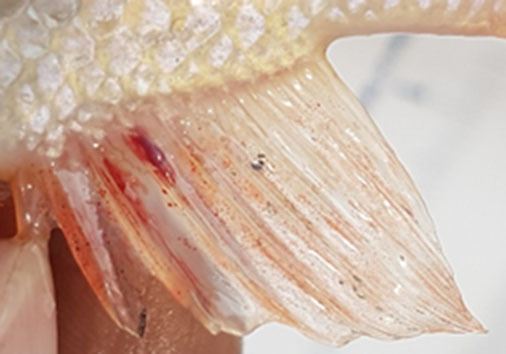

To determine the number of fish with an increase or decrease in K at the end of each experiment, per pond per treatment, the average value of initial K per fish per pond was taken (constant of K per pond), as follows:


$$\begin{array}{l} Final\;K\;per\;fish-constant\;of\;K\;per\;pond \end{array}$$

where the positive values are taken as an increase (0) and the negative values as a decrease (1) in K per fish (Table 2).

The following calculations were performed to determine (29) (Table 2):


$$\begin{array}{l} Incidence\;Welfare\;Indicator\;in\;the\;treatment\;\left(I\;Tx\right)=\left(\frac{nO}{nTx}\right)*100 \end{array}$$

the incidence of the welfare indicator corresponding to each section according to the treatment, where nO: no. individuals that presented the event and, nTx: no. individuals by treatment; observed treatment individuals presenting the event (0 or 1) accordingly to the corresponding index description.


$$\begin{array}{l} Incidence\;of\;the\;same\;Welfare\;Indicator\;in\;the\;rest\;\left(I\right)\\ =\left(\frac{\sum \;nO\;other\;treatments}{\sum \;nTx\;other\;treatments}\right)*100 \end{array}$$

$$\begin{array}{l} Relative\;Risk\;\left(RR\right)=\left(\frac{\%\;I\;Tx}{\%\;I}\right) \end{array}$$

The relative risk was taken with 95% confidence intervals (CI). It is important to note that a RR with a value >1.0 indicates that the factor, in this case, the treatment, represents a risk on the WI evaluated, otherwise, a RR <1.0 indicates a NO risk. If the RR value is equal to 1, then the risk is the same between the groups. The CIs allow determining the lower and upper limits where the real RR value is located, therefore, a lower CI with a value >1.0 indicates a risk at a 95% confidence level.

### WIs Risk Traffic Light

To facilitate the use and practical application of the results reported in this study, a risk traffic light for WIs was designed (Table 3). The ranges of the risk traffic light were determined considering the lowest data of % I Tx, according to the stage and the evaluated welfare indicator (Tables 4–8); if this was too low then the next higher value was used. To consider the lower limit indicator in a situation of moderate risk, the data had to be associated with a RR lower than 0.85 and a present CI in ranges lower than 0.99. As for the upper limit indicator, it was calculated = (% I Tx lower limit * RR constant)/RR; the RR constant was determined as 0.999.

**Table 3:** Suggested risk limits (%) and color for welfare indicator in relation to the individuals present in the culture pond.

*Welfare indicator **%**	**No risk**	**Moderate risk**	**High risk**
**Fingerlings, from 1 g**			
Mortality	<14	14–25	>25
Decreased body weight	<34	34–57	>57
Decreased body condition	<11	11–20	>20
Obvious damage to caudal fin	<6	6–12	>12
Obvious damage to anal fin	<8	8–15	>15
**Juveniles, from 10 g**			
Mortality	<4	4–5	>5
Decreased body weight	<35	35–60	>60
Decreased body condition	<11	11–21	>21
Obvious damage to caudal fin	<6	6–12	>12
Obvious damage to anal fin	<8	8–14	>14
**On-growing 1, from 30 g**			
Mortality	<2	2–13	>13
Decreased body weight	<47	47–58	>58
Decreased body condition	<24	24–53	>53
Obvious damage to caudal fin	<11	11–18	>18
Obvious damage to anal fin	<17	17–19	>19
**On-growing 2, from 65 g**			
Mortality	<2	2–4	>4
Decreased body weight	<50	50–57	>57
Decreased body condition	<34	34–38	>38
Obvious damage to caudal fin	<21	21–30	>30
Obvious damage to anal fin	<15	15–19	>19
**On-growing 3, from 130 g**			
Mortality	<2	2–7	>7
Decreased body weight	<36	36–66	>66
Decreased body condition	<45	45–53	>53
Obvious damage to caudal fin	<29	29–55	>55
Obvious damage to anal fin	<23	23–72	>72

*Welfare Indicator was determined based on the percentage of affected individuals. The ranges of the risk traffic light were determined considering the lowest data (in whole numbers) of % I Tx, according to the stage and the evaluated welfare indicator (Tables 4–8); if this was too low then the next higher value was used. To consider the lower limit indicator in a situation of moderate risk, the data had to be associated with a RR lower than 0.85 and a present CI in ranges lower than 0.99. As for the upper limit indicator, it was calculated = (% I Tx lower limit * RR constant) / RR, and said RR constant was determined as 0.999.

**Table 4:** Contingency table, incidence of mortality, weight reduction, K reduction and caudal and anal fin damage of *O. niloticus* fingerlings according to the feeding rate.

**Records**				**Epidemiological analysis**				
		**nO**	**(%)**	**% I Tx**	**% I**	**RR**	**(CI 95%)**	***p* value**
**Tx**	**n Tx**	**Total**, ***n*** **=** **2,160 (100%)**		**Welfare Indicator**				
		**Death fish**		**Mortality**				
Ufe	540	112	(5.19)	20.74	25.56	0.81	(0.67–0.97)	0.0240
Control	540	74	(3.43)	13.70	27.90	0.49	(0.39–0.61)	0.0000
OfA	540	130	(6.02)	24.07	24.44	0.98	(0.82–1.17)	ns
OfB	540	210	(9.72)	38.89	19.51	1.99	(1.72–2.30)	0.0000
		**Total**, ***n*** **=** **1,634 (100%)**						
		**Weight lost cases**		**Weight reduction**				
Ufe	428	211	(12.9)	49.30	56.97	0.86	(0.77–0.96)	0.0062
Control	466	160	(9.79)	34.33	63.18	0.54	(0.47–0.62)	0.0000
OfA	410	256	(15.6)	62.44	52.45	1.19	(1.08–1.30)	0.0004
OfB	330	271	(16.6)	82.12	48.05	1.70	(1.58–1.84)	0.0000
		**K lost cases**		**K reduction**				
Ufe	428	56	(3.43)	13.08	21.56	0.60	(0.46–0.79)	0.0001
Control	466	52	(3.18)	11.16	22.60	0.49	(0.37–0.65)	0.0000
OfA	410	117	(7.16)	28.54	16.26	1.75	(1.43–2.14)	0.0000
OfB	330	91	(5.57)	27.58	17.25	1.59	(1.29–1.97)	0.0000
		**Damaged caudal fin cases**		**Caudal fin damage**				
Ufe	428	53	(3.24)	12.38	10.20	1.21	(0.89–1.64)	ns
Control	466	28	(1.71)	6.01	12.67	0.47	(0.32–0.69)	0.0001
OfA	410	45	(2.75)	10.98	10.70	1.02	(0.74–1.41)	ns
OfB	330	50	(3.06)	15.15	9.66	1.56	(1.15–2.12)	0.0041
		**Damaged anal fin cases**		**Anal fin damage**				
Ufe	428	36	(2.20)	8.41	16.00	0.52	(0.37–0.73)	0.0001
Control	466	62	(3.79)	13.30	14.30	0.93	(0.70–1.22)	ns
OfA	410	68	(4.16)	16.59	13.15	1.26	(0.97–1.63)	ns
OfB	330	63	(3.86)	19.09	12.73	1.50	(1.15–1.95)	0.0029

Tx, treatment; n Tx, no. fish by treatment; nO, no. observed fish who presented the event (cases); %, proportion of individuals who presented the event (cases) in relation to the total n; p-value calculated by means of X^2^; I Tx: incidence of the variable corresponding to each section according to the treatment, calculated = (nO / nTx)*100; I: incidence of the same variable in the rest of the individuals, calculated = (sum nO of the other treatments/sum nTx of the other treatments)*100; RR: relative risk, calculated = % I Tx / % I; CI, confidence intervals (95%).

*Treatments: Feeding rate in relation to the pond biomass for underfeeding (Ufe, 80%), control (100%), overfeeding A (OfA, 120%), and overfeeding B (OfB, 140%), respectively*.

### Statistical Analysis

To determine the probability of occurrence or not of an event (0 or 1), according to the welfare indicator and index description, being death or, where appropriate, the visible damage observed in the experimental organisms (each dead fish or fish with visible damage is considered an event: nO), the presence/absence data of the welfare indicator were analyzed from an epidemiological approach through a prospective, longitudinal, analytical, experimental study (29). Contingency tables were used to determine the % incidence of the welfare indicators (events) presented in a particular treatment (% I Tx), the % incidence of the welfare indicators presented in the rest of the treatments (% I), the relative risk (RR), and 95% confidence intervals. The independence tests were performed using the Chi-square test (which measures the degree of association or relationship between the treatment and the welfare indicator) (28). The total number of animals per treatment was used to develop the contingency table analysis. A one-way ANOVA was used to analyze the biomass production values, followed by a Duncan test for, bias and kurtosis previously reviewed. To jointly analyze the data of the growth stages, the values were transformed into %, considering the highest value obtained from biomass production as 100%. Statistical program Statgraphics XVI, was used.

## Results

Statistically significant associations (Chi-square test, p <0.05) were observed between the treatments and the welfare indicators (WIs) of mortality, weight reduction, K reduction, and presence of evident damage in caudal and anal fins. In the rest of the WIs evaluated, no statistically significant relationships were observed. In all the stages studied, statistically significant associations were observed between the treatments and the five WIs mentioned (Tables 4–8). The first section of Table 4 is described in detail below to facilitate the reading and understanding of the results of this study. Total n = number of fish that were part of the corresponding WI analysis (mortality), which for on-growing 1 was 2,160 fingerlings; n Tx = number of fish per treatment (540 for each); nO = number of observed fish that presented the WI, in this case death, being 112, 74, 130, and 210 dead fish throughout the experimental period, for the Uf, Control, OfA and OfB treatments, respectively; % = number of WIs in relation to Total n (2,160 = 100%), being 5.19, 3.43, 6.02 and 9.72% according to each treatment; *p* value = statistical significance of the Chi-square test analysis; % I Tx = Incidence of the WI in a particular treatment, it is obtained by performing the following operation: (nO / n Tx) * 100 resulting in 20.74, 13.70, 24.07 and 38.89% for the Ufe, Control, OfA and OfB treatments, respectively; % I = Incidence of the WI in the rest of the treatments, therefore, for the treatment of Ufe, the following operation is obtained: (74 + 130 + 210 / 540 + 540 + 540) * 100 = (414 / 1,620 ) * 100 = 25.56. The RR is the relationship between % I Tx and % I, and RR = 20.74 / 25.56 = 0.81 are obtained for the Ufe treatment, RR = 13.70 / 27.90 = 0.49 for the Control, RR = 24.07 / 24.44 = 0.98 for the treatment OfA, and RR = 38.89 / 19.51 = 1.99 for OfB, respectively. In the following sections of Table 4, which correspond to the rest of the WIs evaluated (weight reduction, K reduction, damage to caudal and anal fins), Total n is the number of fish that survived at the end of the experiment, in this case, and accordingly to the calculation made corresponding to the number of surviving fish (1,634 fish), distributed in the treatments (428, 466, 410, and 330, respectively).

In fingerlings, a RR of 1.99 for the WI mortality is observed in the OfB treatment, this means that the probability of observing dead fish under this risk condition is 1.99 times greater than the mortality probability, in the rest of the treatments (Table 4). Compared to the Control treatment, where a lower RR was observed, the probability of death is 0.49 times the probability of observing dead fish in the other treatments. This means that the treatment factor OfB represents a mortality risk of practically four times, compared to the mortality risk of the Control treatment. With the WI of weight reduction, a RR of 0.54 was observed in Control, and in the OfB of 1.70, which means that there is 3.49 times more risk that the fish lose weight than with the control treatment. In the WI of K reduction, the OfA treatment obtained a RR of 1.75, while the Control treatment a RR of 0.49, with which in the OfA treatment it is up to 3.5 times more likely that the fish will see their body condition reduced than in the Control treatment. The WI of caudal fin damage showed that in the OfB treatment the RR was 1.56, while in the control treatment the RR was 0.47, with which in OfB the fish have up to 3.32 times the risk of injury to the caudal fins than in the control treatment. The anal fin damage WI presented that the RR of OfB was 1.50 and the Ufe treatment of 0.52, which indicates that in OfB the fish have a risk of 2.88 more lesions in the anal fin than in the Ufe treatment.

In treatment OfB a RR of 2.0 for the WI mortality is observed for the juvenile stage (Table 5), meaning that the probability of observing dead fish in the OfB treatment is 2.0 times greater than the probability of mortality in the rest of the treatments. Compared with the Ufe and control treatments, where a lower RR is observed, the probability of death is 0.64 times the probability of observing dead fish in the other treatments. The OfB treatment factor represents a mortality risk of practically 3.12 more, compared to the mortality risk of the Ufe and control treatments. With the WI of weight reduction, the RR of 0.52 was observed in the OfA, and in the Ufe of 1.78, with which there is 3.42 more risk that the fish lose weight than with the OfA. In the WI of K reduction, the Ufe treatment obtained a RR of 1.65, while the OfA treatment a RR of 0.49, with which in the Ufe treatment it is up to 3.36 more likely that the fish will see their body condition reduced than in the OfA treatment. The WI of caudal fin damage showed that in the OfB treatment a RR of 1.42, while in the Control treatment the RR was 0.45, with which in OfB the fish have up to 3.32 times more risk of injury to the caudal fin than in Control. Anal fin damage WI in the OfB treatment presented a RR of 1.49 and in Ufe of 0.52, which indicates that fish in OfB conditions have a 2.88 higher risk of lesions in the anal fin than in Ufe.

**Table 5:** Contingency table, incidence of mortality, weight reduction, K reduction, and caudal and anal fin damage of *O. niloticus* juvenile according to the feeding rate.

**Records**				**Epidemiological analysis**				
		**nO**	**(%)**	**% I Tx**	**% I**	**RR**	**(CI 95%)**	***p* value**
**Tx**	**n Tx**	**Total**, ***n*** **=** **1,200 (100%)**		**Welfare Indicator**				
		**Death fish**		**Mortality**				
Ufe	300	8	(0.67)	2.67	4.11	0.64	(0.30–1.37)	ns
Control	300	8	(0.67)	2.67	4.11	0.64	(0.30–1.37)	ns
OfA	300	11	(0.92)	3.67	3.78	0.97	(0.49–1.89)	ns
OfB	300	18	(1.50)	6.00	3.00	2.00	(1.11–3.57)	0.0179
		**Total**, ***n*** **=** **1,155 (100%)**						
		**Weight lost cases**		**Weight reduction**				
Ufe	292	251	(21.7)	85.96	48.09	1.78	(1.64–1.94)	0.0000
Control	292	140	(12.1)	47.95	60.95	0.78	(0.69–0.89)	0.0001
OfA	289	100	(8.66)	34.60	65.36	0.52	(0.44–0.62)	0.0000
OfB	282	175	(15.1)	62.06	56.24	1.10	(0.99–1.22)	ns
		**K lost cases**		**K reduction**				
Ufe	292	83	(7.19)	28.42	17.15	1.65	(1.31–2.09)	0.0000
Control	292	37	(3.20)	12.67	22.48	0.56	(0.40–0.78)	0.0003
OfA	289	33	(2.86)	11.42	22.86	0.49	(0.35–0.70)	0.0000
OfB	282	78	(6.75)	27.66	17.53	1.57	(1.24–2.00)	0.0002
		**Damaged caudal fin cases**		**Caudal fin damage**				
Ufe	292	34	(2.94)	11.64	10.78	1.08	(0.74–1.56)	ns
Control	292	17	(1.47)	5.82	12.75	0.45	(0.27–0.74)	0.0011
OfA	289	36	(3.12)	12.46	10.51	1.18	(0.82–1.70)	ns
OfB	282	40	(3.46)	14.18	9.97	1.42	(1.00–2.01)	0.0490
		**Damaged anal fin cases**		**Anal fin damage**				
Ufe	292	24	(2.08)	8.22	15.76	0.52	(0.34–0.78)	0.0013
Control	292	38	(3.29)	13.01	14.14	0.92	(0.65–1.29)	ns
OfA	289	46	(3.98)	15.92	13.16	1.20	(0.88–1.65)	ns
OfB	282	52	(4.50)	18.44	12.37	1.49	(1.10–2.01)	0.0103

Tx, treatment; n Tx, no. fish by treatment; nO, no. observed fish who presented the event (cases); %, proportion of individuals who presented the event (cases) in relation to the total n; p-value calculated by means of X^2^; I Tx, incidence of the variable corresponding to each section according to the treatment, calculated = (nO / nTx)*100; I, incidence of the same variable in the rest of the individuals, calculated = (sum nO of the other treatments/sum nTx of the other treatments)*100; RR: relative risk, calculated = % I Tx / % I; CI, confidence intervals (95%).

*Treatments: Feeding rate in relation to the pond biomass for underfeeding (Ufe, 80%), control (100%), overfeeding A (OfA, 120%), and overfeeding B (OfB, 140%), respectively*.

For on-growing 1 (Table 6), a RR of 30 for WI mortality is observed in the OfB treatment, this means that the probability of observing dead fish for the OfB treatment is 30 times greater than the probability of mortality in the rest of the treatments. Compared with the Ufe and control treatments, where a lower RR is observed, the probability of death is 0.07 times the probability of observing dead fish in the other treatments. This means that the OfB treatment factor represents a mortality risk of practically 428.57 times more, compared to the mortality risk of the Ufe and Control treatments. With the WI of weight reduction, the RR of 0.73 was observed in the Control, and in the Ufe it was 1.61, with which there is 2.20 more risk that the fish lose weight than in the Control. In the WI of K reduction, the control and OfA treatments obtained a RR of 1.28, while the Ufe treatment had a RR of 0.41, with which in the Ufe treatment they are up to 3.12 times more likely that the fish will suffer reduced bodily condition than in the OfA treatment. The WI of caudal fin damage showed that in the control treatment a RR of 0.55, with which in Control the fish have up to 3.32 times less risk of injury to the caudal fin than in the rest of the treatments, which did not present differences. Anal fin damage WI did not present significant RR in any treatment.

**Table 6:** Contingency table, incidence of mortality, weight reduction, K reduction, and caudal and anal fin damage of on-growing 1 of *O. niloticus* according to the feeding rate.

**Records**				**Epidemiological analysis**				
		**nO**	**(%)**	**% I Tx**	**% I**	**RR**	**(CI 95%)**	***p* value**
**Tx**	**n Tx**	**Total**, ***n*** **=** **1,200 (100%)**		**Welfare Indicator**				
		**Death fish**		**Mortality**				
Ufe	300	1	(0.08)	0.33	4.78	0.07	(0.01–0.50)	0.0004
Control	300	1	(0.08)	0.33	4.78	0.07	(0.01–0.50)	0.0004
OfA	300	2	(0.17)	0.67	4.67	0.14	(0.03–0.58)	0.0014
OfB	300	40	(3.33)	13.33	0.44	30.0	(10.8–83.1)	0.0000
		**Total**, ***n*** **=** **1,156 (100%)**						
		**Weight lost cases**		**Weight reduction**				
Ufe	299	246	(21.2)	82.27	50.99	1.61	(1.48–1.75)	0.0000
Control	299	140	(12.1)	46.82	63.36	0.73	(0.64–0.84)	0.0000
OfA	298	147	(12.7)	49.33	62.47	0.79	(0.69–0.89)	0.0001
OfB	260	150	(12.9)	57.69	59.49	0.97	(0.86–1.09)	ns
		**K lost cases**		**K reduction**				
Ufe	299	72	(6.23)	24.08	58.5	0.41	(0.33–0.50)	0.0000
Control	299	178	(15.4)	59.53	46.2	1.28	(1.14–1.44)	0.0001
OfA	298	177	(15.3)	59.40	46.2	1.28	(1.14–1.44)	0.0001
OfB	260	147	(12.7)	56.54	47.6	1.18	(1.04–1.34)	0.0117
		**Damaged caudal fin cases**		**Caudal fin damage**				
Ufe	299	54	(4.67)	18.06	16.5	1.09	(0.82–1.44)	ns
Control	299	32	(2.77)	10.70	19.1	0.55	(0.39–0.79)	0.0008
OfA	298	56	(4.84)	18.79	16.3	1.15	(0.87–1.52)	ns
OfB	260	54	(4.67)	20.77	15.8	1.31	(0.98–1.73)	ns
		**Damaged anal fin cases**		**Anal fin damage**				
Ufe	299	54	(4.67)	18.06	19.37	0.93	(0.70–1.23)	ns
Control	299	65	(5.62)	21.74	18.09	1.20	(0.92–1.55)	ns
OfA	298	56	(4.84)	18.79	19.11	0.98	(0.74–1.29)	ns
OfB	260	45	(3.89)	17.31	19.53	0.88	(0.65–1.19)	ns

Tx, treatment; n Tx, no. fish by treatment; nO, no. observed fish who presented the event (cases); %, proportion of individuals who presented the event (cases) in relation to the total n; p-value calculated by means of X^2^; I Tx, incidence of the variable corresponding to each section according to the treatment, calculated = (nO / nTx)*100; I, incidence of the same variable in the rest of the individuals, calculated = (sum nO of the other treatments/sum nTx of the other treatments)*100; RR: relative risk, calculated = % I Tx / % I; CI, confidence intervals (95%).

*Treatments: Feeding rate in relation to the pond biomass for underfeeding (Ufe, 80%), control (100%), overfeeding A (OfA, 120%), and overfeeding B (OfB, 140%), respectively*.

For on-growing 2 (Table 7), we observed a RR of 8.50 for the WI mortality in the OfB treatment meaning that the probability of observing dead fish for the OfB treatment is 8.50 times greater than the probability of mortality in the rest of the treatments, where no significant RR was reported. With the WI of weight reduction, the RR of 0.79 was observed in the Control, and in the OfA of 1.14, with which there is 1.44 more risk that the fish lose weight than in the control. In the WI of K reduction, the Ufe treatment obtained a RR of 1.19, while the control treatment a RR of 0.82, with which in the Ufe treatment they are up to 1.45 more likely that the fish will see their body condition reduced than in the OfA treatment. The WI of caudal fin damage showed that in the Ufe treatment a RR of 1.40, while in the OfA treatment the RR was 0.62, with which in OfB the fish have up to 2.25 times the risk of injury to the caudal fins than in control. Anal fin damage WI showed that the control RR was 1.65 and that of Ufe was 0.74, which indicates that in Control the fish have a risk of 2.22 more of presenting lesions in the anal fin than in Ufe.

**Table 7:** Contingency table, incidence of mortality, weight reduction, K reduction, and caudal and anal fin damage of on-growing 2 of *O. niloticus* according to the feeding rate.

**Records**				**Epidemiological analysis**				
		**nO**	**(%)**	**% I Tx**	**% I**	**RR**	**(CI 95%)**	***p* value**
**Tx**	**n Tx**	**Total**, ***n*** **=** **1,200 (100%)**		**Welfare Indicator**				
		**Death fish**		**Mortality**				
Ufe	300	3	(0.25)	1.00	2.22	0.45	(0.13–1.50)	ns
Control	300	3	(0.25)	1.00	2.22	0.45	(0.13–1.50)	ns
OfA	300	0	(0.00)	0.00	2.56	0.00	n/a	0.0052
OfB	300	17	(1.42)	5.67	0.67	8.50	(3.38–21.8)	0.0000
		**Total**, ***n*** **=** **1,177 (100%)**						
		**Weight lost cases**		**Weight reduction**				
Ufe	297	175	(14.8)	58.94	60.00	0.98	(0.88–1.09)	ns
Control	297	149	(12.6)	50.17	62.95	0.79	(0.70–0.90)	0.0001
OfA	300	198	(16.8)	66.00	57.58	1.14	(1.03–1.26)	0.0103
OfB	283	181	(15.3)	63.96	58.39	1.09	(0.98–1.21)	ns
		**K lost cases**		**K reduction**				
Ufe	297	135	(11.4)	45.45	38.0	1.19	(1.02–1.38)	0.0246
Control	297	102	(8.67)	34.34	41.8	0.82	(0.68–0.97)	0.0229
OfA	300	117	(9.94)	39.00	40.2	0.96	(0.82–1.14)	ns
OfB	283	116	(9.86)	40.99	36.6	1.03	(0.88–1.21)	ns
		**Damaged caudal fin cases**		**Caudal fin damage**				
Ufe	297	113	(9.60)	38.05	27.0	1.40	(1.17–1.68)	0.0003
Control	297	91	(7.73)	30.64	29.5	1.03	(0.84–1.26)	ns
OfA	300	62	(5.27)	20.67	32.9	0.62	(0.49–0.79)	0.0001
OfB	283	85	(7.22)	30.04	29.7	1.00	(0.82–1.23)	ns
		**Damaged anal fin cases**		**Anal fin damage**				
Ufe	297	46	(3.91)	15.49	20.8	0.74	(0.55–1.00)	0.0457
Control	297	82	(6.97)	27.61	16.7	1.65	(1.30–2.09)	0.0000
OfA	300	49	(4.16)	16.33	20.5	0.79	(0.59–1.06)	ns
OfB	283	52	(4.42)	18.37	19.8	0.92	(0.70–1.22)	ns

Tx, treatment; n Tx, no. fish by treatment; nO, no. observed fish who presented the event (cases); %, proportion of individuals who presented the event (cases) in relation to the total n; p-value calculated by means of X^2^; I Tx, incidence of the variable corresponding to each section according to the treatment, calculated = (nO / nTx)*100; I, incidence of the same variable in the rest of the individuals, calculated = (sum nO of the other treatments/sum nTx of the other treatments)*100; RR: relative risk, calculated = % I Tx / % I; CI, confidence intervals (95%).

*Treatments: Feeding rate in relation to the pond biomass for underfeeding (Ufe, 80%), control (100%), overfeeding A (OfA, 120%), and overfeeding B (OfB, 140%), respectively*.

For the on-growing 3 (Table 8), a RR of 9.27 for WI mortality is observed in the OfB treatment meaning that the probability of observing dead fish for the OfB treatment is 9.27 times greater than the probability of mortality in the rest of the treatments. Compared with the control treatment, where a lower RR is observed, the probability of death is 0.21 times that of observing dead fish in the other treatments. This means that the treatment factor OfB represents a mortality risk of practically 44.1 more, compared to the mortality risk of the control treatment. With the WI of weight reduction, a RR of 0.49 was observed in the Ufe, and the OfA and OfB both observed a 1.46 RR, with which there is 2.97 more risk that the fish lose weight than in the Ufe. In the WI of K reduction, the OfA treatment obtained a RR of 1.48, while the Ufe treatment a RR of 0.76, which means the OfA is up to 1.94 more likely that the fish will see their body condition reduced than in the Ufe treatment. The WI of caudal fin damage showed that in the OfA treatment a RR of 1.39, while in the OfB treatment the RR was 0.48, with which in OfA the fish have up to 2.89 times the risk of tail fin injury than in OfB. Anal fin damage WI showed that the RR of OfB was 0.29, while in the others the RR was the same. It should be noted that in these last two WIs where OfB came out with a lower RR than the other treatments, it was due to the poor quality of the water, which affected the fish behavior.

**Table 8:** Contingency table, incidence of mortality, weight reduction, K reduction, and caudal and anal fin damage of on-growing 3 of *O. niloticus* according to the feeding rate.

**Records**				**Epidemiological analysis**				
		**nO**	**(%)**	**% I Tx**	**% I**	**RR**	**(CI 95%)**	***p* value**
**Tx**	**n Tx**	**Total**, ***n*** **=** **720 (100%)**		**Welfare Indicator**				
		**Death fish**		**Mortality**				
Ufe	180	7	(0.97)	3.89	7.04	0.55	(0.25–1.21)	ns
Control	180	3	(0.42)	1.67	7.78	0.21	(0.06–0.68)	0.0034
OfA	180	1	(0.14)	0.56	8.15	0.06	(0.009–0.49)	0.0003
OfB	180	34	(4.72)	18.89	2.04	9.27	(4.79–17.91)	0.0000
		**Total**, ***n*** **=** **675 (100%)**						
		**Weight lost cases**		**Weight reduction**				
Ufe	173	62	(9.19)	35.84	72.91	0.49	(0.39–0.60)	0.0000
Control	177	95	(14.0)	53.67	66.87	0.80	(0.69–0.93)	0.0017
OfA	179	148	(21.9)	82.68	56.45	1.46	(1.32–1.62)	0.0000
OfB	146	123	(18.2)	84.25	57.66	1.46	(1.32–1.61)	0.0000
		**K lost cases**		**K reduction**				
Ufe	173	77	(11.4)	44.51	58.37	0.76	(0.63–0.91)	0.0016
Control	177	87	(12.8)	49.15	56.83	0.86	(0.73–1.02)	ns
OfA	179	129	(19.1)	72.07	48.59	1.48	(1.30–1.68)	0.0000
OfB	146	77	(11.4)	52.74	55.39	0.95	(0.80–1.13)	ns
		**Damaged caudal fin cases**		**Caudal fin damage**				
Ufe	173	51	(7.56)	29.48	30.28	0.97	(0.74–1.27)	ns
Control	177	60	(8.89)	33.90	28.71	1.18	(0.92–1.51)	ns
OfA	179	68	(10.0)	37.99	27.22	1.39	(1.10–1.76)	0.0071
OfB	146	24	(3.56)	16.44	33.84	0.48^*^	(0.33–0.71)	0.0000^*^
		**Damaged anal fin cases**		**Anal fin damage**				
Ufe	173	40	(5.93)	23.12	18.72	1.23	(0.89–1.71)	ns
Control	177	41	(6.07)	23.16	18.67	1.24	(0.89–1.71)	ns
OfA	179	43	(6.37)	24.02	18.35	1.30	(0.95–1.80)	ns
OfB	146	10	(1.48)	6.85	23.44	0.29^*^	(0.15–0.54)	0.0000^*^

Tx, treatment; n Tx, no. fish by treatment; nO, no. observed fish who presented the event (cases); %, proportion of individuals who presented the event (cases) in relation to the total n; p-value calculated by means of X^2^; I Tx, incidence of the variable corresponding to each section according to the treatment, calculated = (nO / nTx)*100; I, incidence of the same variable in the rest of the individuals, calculated = (sum nO of the other treatments/sum nTx of the other treatments)*100; RR: relative risk, calculated = % I Tx / % I; CI, confidence intervals (95%).

* *False positive, as an effect of the high mortality that occurred in said treatment*.

*Treatments: Feeding rate in relation to the pond biomass for underfeeding (Ufe, 80%), control (100%), overfeeding A (OfA, 120%), and overfeeding B (OfB, 140%), respectively*.

**Figure 1:**
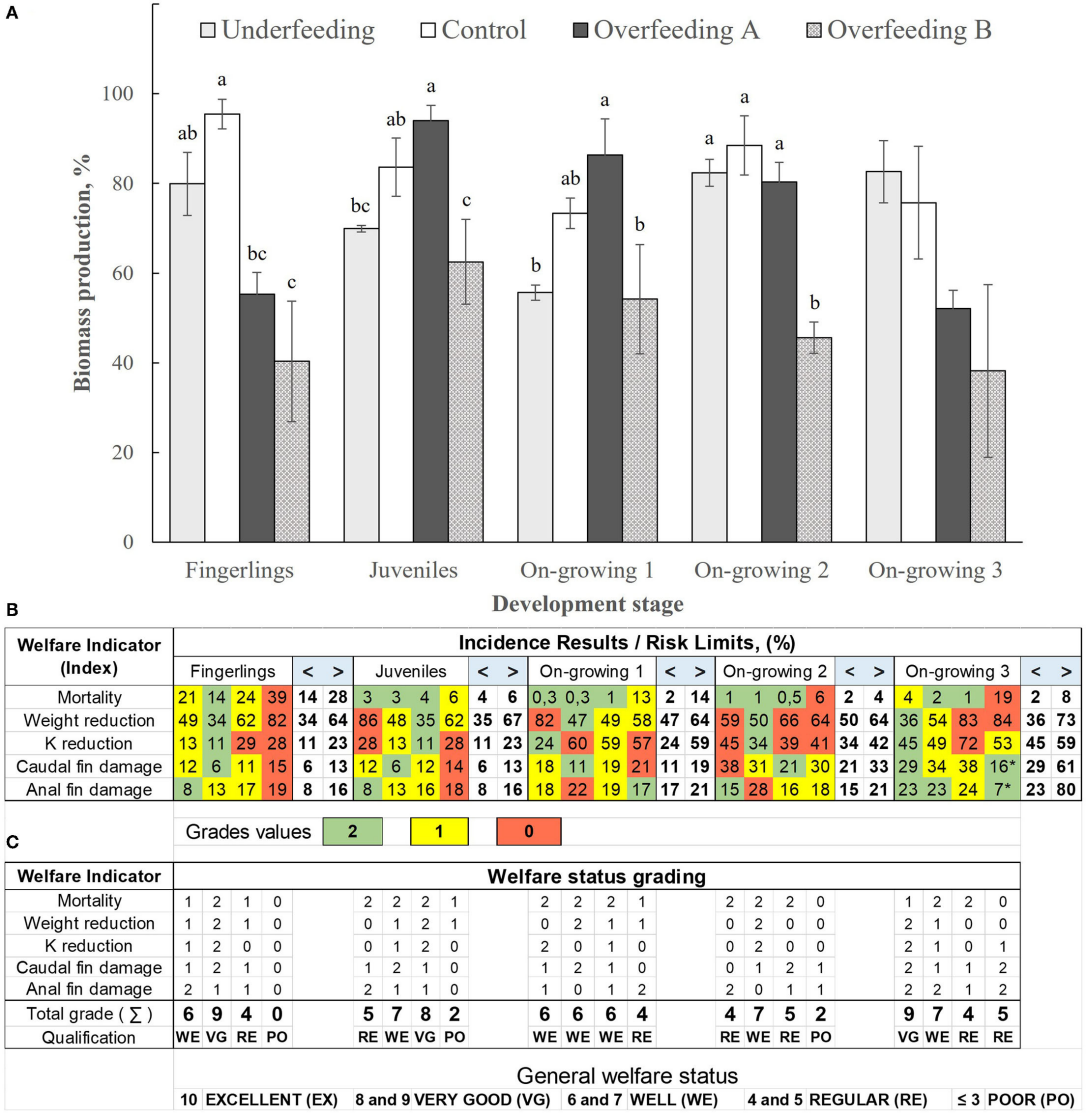
**(A)** Biomass production per pond per treatment (means ± SE) in the 5 experiments carried out according to the treatments: underfeeding (80%), control (100%), overfeeding A (120%), and overfeeding B (140%). **(B)** Each cell in color is the value resulting from the % I Tx of the epidemiological analysis (integer) according to the welfare indicator of each treatment (from Tables 4–8). White cells are risk limits (%) for welfare indicators in relation to the individuals present in the culture pond of each treatment (from Table 3; < and >). **(C)** The application of the proposed epidemiological traffic light and the qualification of the welfare state by treatment according to the evaluated indicators. Color traffic lights are categorized as Green (2 points), Yellow (1 point), and Red (0 points). The value of the evaluated indicators added (summative) to obtain a comprehensive/overall qualification (fingerlings to on growing 3) of the welfare state according: Excellent (EX: 10 points), Very Good (VG: 8 and 9 points), Well (WE: 6 and 7 points), Regular (RE: 4 and 5 points) and Poor (PO: ≤ 3 points). *False positive, as an effect of the high mortality occurred in said treatment. Means ± SE.

The registered water quality values during the study are in Table 9. The biomass production data per pond concerning the treatments are in Figure 1. In the fingerling stage, we observed highly significant differences between the treatments (*p* < 0.01). The Control treatment showed the highest value (95.47%), followed by Ufe (79.90%), and the lowest value in OfB (40.33%). In the juvenile phase, there were significant differences between feeding rates (*p* < 0.05). OfA being the maximum value recorded (94.03%) after the Control treatment (83.63%), the lowest biomass production was observed in OfB. In the group of fish with on-growing 1, significant statistical differences were found between the treatments (*p* < 0.05), with OfA where the highest production occurred and the lowest in the overfeeding treatments (OfA and OfB). In on-growing 2, highly significant differences were observed between treatments (*p* < 0.01). In the control, Ufe and OfA, the highest values were observed (88.50, 82.33, and 80.33%, respectively), and the lowest value was in the OfB treatment (45.60%). Regarding on-growing 3 phase, the ANOVA analysis did not show significant statistical differences between the treatments, however, in Figure 1 it can be observed that the highest productions were recorded in the Ufe and control treatments, and the lowest in OfA and OfB. In the lower part of Figure 1, the application of the proposed epidemiological traffic light and the qualification of welfare by treatment according to the evaluated indicators can be observed. To determine the degree of welfare, the color of the traffic light was categorized as follows:

Green = 2 points
Yellow = 1 point
Red = 0 points

**Table 9:** Water quality values during the study.

**Treatments**	**Underfeeding**	**Control**	**Overfeeding A**	**Overfeeding B**
**Temperature (°C)**				
Fingerlings	26.4 ± 1.8	26.2 ± 2.1	26.4 ± 1.8	28.7 ± 2.1
Juveniles	25.3 ± 1.5	25.0 ± 1.8	25.2 ± 1.4	25.6 ± 1.6
On-growing 1	24.9 ± 1.2	24.5 ± 1.7	24.6 ± 1.3	25.1 ± 1.6
On-growing 2	26.7 ± 1.5	25.3 ± 1.8	25.4 ± 1.4	25.8 ± 2.2
On-growing 3	23.6 ± 1.6	23.2 ± 1.6	23.4 ± 1.4	23.1 ± 2.1
**Disolved Oxigen (mg L** ^**−1**^ **)**				
Fingerlings	5.5 ± 0.5	4.9 ± 0.6	4.7 ± 0.7	3.5 ± 0.6
Juveniles	5.4 ± 0.6	4.9 ± 0.7	4.7 ± 0.8	3.3 ± 1.1
On-growing 1	5.8 ± 0.8	5.2 ± 0.8	5.1 ± 0.7	3.7 ± 0.9
On-growing 2	5.5 ± 0.6	4.9 ± 0.6	4.7 ± 0.8	3.5 ± 0.9
On-growing 3	5.7 ± 0.8	5.4 ± 0.9	4.6 ± 1.0	3.7 ± 1.0
**Electrical Conductivity (mS/cm** ^**3**^ **)**				
Fingerlings	699.3 ± 31.2	725.6 ± 39.5	863.4 ± 44.7	896.7 ± 50.8
Juveniles	792.4 ± 39.2	790.5 ± 46.0	815.9 ± 45.6	813.3 ± 48.2
On-growing 1	777.0 ± 37.9	760.9 ± 107.9	791.2 ± 44.7	808.2 ± 52.2
On-growing 2	919.7 ± 137.2	952.6 ± 153.9	978.0 ± 182.4	1070.9 ± 198.1
On-growing 3	947.9 ± 221.0	943.1 ± 209.8	921.7 ± 198.4	951.7 ± 235.8
${\text{NH}}_{4}^{+}$ **(mg L**^**−1**^**)**				
Fingerlings	0.1 ± 0.1	0.3 ± 0.2	0.3 ± 0.2	0.7 ± 0.1
Juveniles	0.3 ± 0.2	0.4 ± 0.4	0.8 ± 0.6	0.9 ± 0.3
On-growing 1	0.5 ± 0.1	0.7 ± 0.2	0.8 ± 0.7	0.9 ± 0.1
On-growing 2	0.5 ± 0.1	0.6 ± 0.4	0.8 ± 0.4	0.9 ± 0.3
On-growing 3	0.2 ± 0.1	0.2 ± 0.2	0.3 ± 0.2	0.3 ± 0.2
**pH**				
Fingerlings	7.2 ± 0.1	7.3 ± 0.1	7.1 ± 0.2	7.5 ± 0.2
Juveniles	7.5 ± 0.2	7.3 ± 0.2	7.3 ± 0.1	7.3 ± 0.2
On-growing 1	7.3 ± 0.1	7.2 ± 0.1	7.2 ± 0.1	7.2 ± 0.1
On-growing 2	7.3 ± 0.1	7.2 ± 0.1	7.3 ± 0.1	7.3 ± 0.1
On-growing 3	7.4 ± 0.1	7.3 ± 0.1	7.3 ± 0.1	7.3 ± 0.1

*Means ± SD*.

Subsequently, the value of the evaluated indicators was added to obtain a comprehensive qualification (fingerlings to on growing 3) of the welfare state according to the following:

10 points = Excellent (EX)
8 and 9 points = Very Good (VG)
6 and 7 points = Well (WE)
4 and 5 points = Regular (RE)
≤ 3 points = Poor (PO)

## Discussion

To the authors' knowledge, this is the first report on the analysis of welfare indicators (WIs) using an epidemiological population approach tools at various stages of the tilapia life cycle. To determine if the production conditions that arise because of feed management, in particular the feeding rate used, can be a risk factor on the welfare status of a fish population in a defined time. Fish welfare is an issue that concerns fish farmers in daily practice, whose objective is to be able to produce organisms properly and follow the correct guidelines for growing this species (8, 14), carrying out responsible and sustainable management. Currently, the consumer is willing to pay an additional price for fish products from farms that are identified with quality and welfare standards (40), therefore, it is important to identify according to the species, which WI's can be used as a standard on laboratories and farms, that is, as laboratory welfare indicators or operational welfare indicators (10).

In the fingerling stage, the water quality indicators were observed within the optimal ranges for tilapia cultivation, except ${\text{NH}}_{4}^{+}$ in the OfB that already presented levels higher than those recommended for the species (41, 42). In the juvenile stages, on-growing 1 and on-growing 2, ${\text{NH}}_{4}^{+}$ was recorded above the optimal values lower than 0.70 mg L^−1^ (41, 42) in the OfA and OfB treatments and the control treatment in the on-growing 1. In on-growing 3, all the water indicators were optimal. High feeding rates rapidly affect water quality. As a fish health indicator, this is important, as ponds exceeding the limits for ammonium, nitrates, and nitrites compromise the development and survival of the organisms (1, 43–45). In addition, a higher concentration of nitrogenous products in the system can favor higher productivity in the pond, which is usually accompanied by a decrease in dissolved oxygen in the water during the early morning hours (46). According to the results, in fingerlings a greater growth was observed using the control feeding rate (8% with 49% CP), in addition to this, that treatment, it was where the best values of WIs were observed. It is important to mention that the higher mortality and lower production occurred in the overfeeding treatments, showing that the fingerlings are very sensitive to water conditions (41). Feed management directly impacts the quality of the culture water (47) as can be seen in the results presented here, the concentration of ${\text{NH}}_{4}^{+}$ and dissolved solids in pond water increases as more food to the fish. Contrarily, in the following two stages (juveniles and on-growing 1), the highest biomass obtained was registered when the fish had an overfeeding of 20% greater than that indicated for the species. It should be noted that it is recommended that tilapia diets decrease the concentration of crude protein as the size of the fish increases (48, 49) because young animals need nutrients steadily, the feeding rate is also higher, as well as the frequency of feeding the diet. As the fish grow, the feeding rates and frequencies decrease, since their metabolism is more efficient to take advantage of the nutrients in the feed (20, 50, 51). Although the commercial diets used in the present study, for the juvenile and on-growing 1, are similar in the contribution of protein and energy, the recommended rate (5 and 4%, respectively) (22), could not be covering the nutritional requirements of the species, for which, and as a result of this study, a rate between 5 and 6% (with 47–48% of CP) and 4 and 4.8% is suggested from the point of view of obtaining biomass (with 44–45% CP), respectively. These values are like those studied by El-Dakar et al. (52) in fingerlings and juveniles of hybrids of *O. niloticus* and *O. mossambicus*. In addition, it was in these two growth stages where greatest the values in % of fish with lesions were recorded, in a moderate risk range of welfare indicators evaluated.

In on-growing 2, the best growths were observed in the control treatment, as were the WIs, except in the incidence of lesions in the anal fin, which suggests a change in behavior concerning age. Tilapia is a cichlid, so aggressiveness is part of its normal behavior, and one of the targets of social injuries is the anal fins (53). Social lesions also occur in other types of fish, such as Siluridae, where the objective is to attack the flanks (54). The purpose of these injuries is to warn other members of their species about the hierarchy and occupation of space, not to hurt (53), however, these injuries can be aggravated if the environment of culture is not suitable (55). Regarding on-growing 3, the highest biomass obtained was recorded in the Ufe treatment, indicating a better use of the feed nutrients (20, 50, 51). The survival of tilapia depends on the type of facilities available, the temperature, the feeding, and the age of the culture (42). In RAS, the survival of the fish is greater, since the conditions are more stable than in open-air cultivation (earthen ponds), as there is a lower proportion of incidences due to the action of predators and diseases (1, 56). In this type of system, 0 to 20% mortality has been observed (17, 32), results like those observed in the OfB treatments in the present study.

The decrease in fish weight is one of the main concerns among aquaculturists; it is very difficult to observe growth with the naked eye but, feasible to follow the development of the pond over time, during cultivation, in the unfolds and pond changes. According to Rey et al. (57), the weight and the comparison of this value must be done by pond because each cage or pond behaves differently. They have their environment. The most advisable action is periodacally to monitor the population and consider the average weight of the pond, instead of making comparisons against databases, especially if they are from regions with different conditions. Therefore, using indicators such as the incidents proposed in this study will allow monitoring of the physiological condition and welfare of the organisms during the production cycle. To identify possible higher-risk situations and make timely and consistent decisions with each stage of growth. Body condition (K) is a variable factor and fluctuates throughout the life cycle of the fish. It is difficult to define exact values that are indicative of reduced welfare, but <0.9 is usually indicative of emaciation (21). Body condition in fish has been used as a productive indicator, making it possible to measure fish growth over time. It has also been mentioned that it is an indicator of welfare in salmon (10), and in perch has been compared between manual and automatic feeding methods (34). What is absent in the previous studies is that there is no average initial body condition. So, it is not known how much it increased or decreased over the experiment time, a fact that is obtained in the present study and that was used as a comparative in the final body condition of the individuals. Thus, body condition can be used as a measurable indicator of welfare, particularly if it is based on the premise that fish entering a culture stage are expected to increase or at least maintain their body condition.

In the present study, was determined that the relationship between the incidence of tilapia caudal fin damage arises because of the used feeding rate. When feed is restricted, frequent and significant damage to fins caused by aggression from dominant individuals is observed, in addition to a decrease in body condition (33). It has been reported that feeding management directly affects the integrity of the fins of *Perca fluviatilis* L. (34) and *Salmo salar* (33), depending on the stage of the culture. However, these studies evaluated the relationship between the feeding management factor and the damage caused to the fins but, did not determine whether feeding management is a risk factor related to the proportion of animals that present evident damage to the fins. The fish fins are part of their anatomy since these limbs allow them to move in their environment (58). The fins are prone to damage their integrity can be affected by various elements, both biotic and abiotic, which can be detrimental to welfare. These factors include population density (59, 60), presence of disease (61), abrasions from pond and cage surfaces (62), aggressions among members of the population (63, 64), feeding management (65–67) and poor water quality, which is related to directly to various circumstances such as low dissolved oxygen concentration, and high concentration of NH4+ and dissolved solids (55, 68). One of the most important fins for fish is the caudal fin since they are propelled by this member and allow them to measure the force with which they move (58).

According to a study in salmon (33), where two experimental groups were used: control and with food restriction, it is reported that 7.5 and 12.5% of the population presented damage, for the control and food restriction, respectively. It should be mentioned that, at first glance, it can infer that there is an effect of the treatment on the number of animals that presented damage. Analyzing the reported data using the Chi-square test, there is no statistically significant relationship between each treatment and the number of observed events. In this case, fish with dorsal fin damage (p = 0.4561). It is important to emphasize that the authors mentioned that, in the food restriction treatment, fins with a greater degree of damage were observed than those that were presented in the control treatment. Although in the present study, damage to the dorsal fin was not recorded in the tilapias. According to the risk traffic light proposed here (Table 8), fish weighing 66 g (on-growing 2 in tilapia), similar to those in the study by Cañon-Jones et al. (33) (initial weight 61.7 ± 6.4 g), is considered a risky situation when from 15 and 21% of the population presented damage to the anal and caudal fins, respectively, values higher than those observed in salmon (7.5 and 12% ), which is probably why the Chi-square test did not show a statistically significant relationship, which is consistent with the present study.

WIs have been determined in various species (10, 15), and are usually specific to the physiological or life stage (15) and the type of culture system used (10). The results obtained in the present study indicate that the 5 experiments allow us to see, as a whole, the effect of the feeding rate in the different growth stages of *O. niloticus*. By integrating the risk limits (%) in a final assessment (Excellent, Very Good, Well, Regular and Poor; Figure 1), a congruence is observed in the condition or general welfare status of the fish analyzed through the monitoring of survival, weight, Fulton index and damage to fins (anal and caudal). Although, in all the stages these same indicators were found where statistically significant relationships were observed, the level of risk changes due to the interaction between the growth stage and the feeding treatment. Mortality, for example, reaches a high risk (cells in red Figure 1), in the overfeeding treatment B for the phases of fingerlings, and on-growing 2 and 3, and only alert or moderate risk (cells yellow), for the juvenile and on-growing 1 phase. This indicates that, despite being the same species, the fish in the early growth phases behave differently in their development and physiology of the amount, quality, and characteristics of the food provided (20, 25, 47, 50).

Gutierrez-Rabadan et al. (15), established WI's in the lumpfish (*Cyclopterus lumpus*), using two experimental groups: animals that were in the hatchery and those in cages, with 60 and 35 animals, respectively. Of the fish in the first group (*n* = 60), 31 presented caudal fin damage, 32 with pelvic fin damage, and in the second group (*n* = 35), 6 fish with tail fin damage and 6 with fin injuries. Proportionally, it is reported that 52 and 53% of the population present damage to the caudal and pelvic fin respectively. In the hatchery, and 6 and 6% to the caudal and anal fin respectively, when fish are kept in cages. When analyzing these same data using the Chi-square test, we observe that there is a statistically significant relationship between the treatment (hatchery) and the number of observed events (fish with damage to the fins) (*p* = 0.0009 for caudal fin; *p* = 0.0005 for pelvic fin). Fin damage can result from aggression but also stress (60). Also, can cause detrimental effects on growth and survival by increasing susceptibility to opportunistic infections. Although in the tilapias of the present study, damage to the pelvic fins was not recorded. According to the risk traffic light proposed here (Table 8), in fish weighing 5 to 152 g (growth stages 2 to 5 in tilapias in this study), like those from Gutierrez-Rabadan et al. (15) (weight range 5–152 g), is considered a risky situation when 12% of the population show damage to the anal and caudal fins, respectively, a lower value than those observed in lumpfish (52 and 53%), which is probably why the Chi-square test showed a statistically significant relationship. When calculating the RR (5.16 for tail fin with CI of 1.87–14.24; 3.11 for pelvic fin with CI of 1.44–6.69), it is observed that culture management directly impacts fish welfare indicators, that is, it is a risk factor to the population.

The integration of the indicators allows qualifying of the general condition of wellbeing obtained in each treatment. Thanks to this analysis tool are possible to observe that the most appropriate feeding protocol depends on the phase of the life cycle of the tilapia. In the present study, the control treatment followed the recommendation proposed for the species (22). Using this regimen, the best growth was obtained in fingerlings and on-growing 2 phases which coincide with the best general welfare state rating. (Very well and well, respectively). However, for the juvenile and on-growing 1 phase, the best biomass production, and general welfare rating were obtained with the overfeeding treatment A (120%), indicating a higher nutritional requirement, particularly protein, in these phases (49). In practice, this form of data visualization and analysis could be useful for making and correcting decisions in crop management and planning an adequate feeding regimen, thanks to the evaluation of the general welfare status of the animals with operational indicators.

In a high-risk productive activity such as aquaculture, increasing efficiency and reducing risks are permanent goals. Usage of operational welfare indicators offers the use of easily obtained biological information. Allowing the identification of “red flags” promptly to avoid breaks in the production cycles. The epidemiological analysis showed its potential application and the methodology to obtain specific alarm values related to the characteristics of each farm (growth stage, feeding amount, the season of the year, etc.). Generating animal welfare programs proposes the opportunity to systematically test the inputs for production (genetic lines, type of feed, tolerance to environmental factors, etc.) and increase efficiency with a focus on constant improvement.

## Conclusions and Animal Welfare Implications

To have information that allows people responsible for tilapia production, either on the farm or in experiments to make prompt decisions and evaluations is a must. Mortality incidence, weight reduction, K reduction, and damage to caudal and anal fins could be used as laboratory welfare indicators or operational welfare indicators in the cultivation of *O. niloticus* applying an epidemiological approach. The feeding rate used directly affects production and the welfare indicators, and in a different way depending on the growth phase. As a result of this study, the epidemiological approach seems to be a valuable tool for production. The proposed risk traffic light method could have great potential, with the suggested limits for WI's concerning the individuals present in the culture pond, allowing progressive evaluation and decision-making to correct risky situations that arise.

## Data Availability Statement

The datasets presented in this article are not readily available because The dataset is part of ongoing research and is still being used to complete it. Requests to access the datasets should be directed to ar.martinez@ugto.mx.

## Ethics Statement

The animal study was reviewed and approved by Institutional Committee of Bioethics in Research of the University of Guanajuato (code: CIBIUG-A59-2020).

## Author Contributions

LF-G: investigation, data obtaining, data analysis, and writing—original draft preparation. JC-C: investigation and data collection. CP-J: data analysis, writing, and partial economical support. PA-R: data analysis and writing—review and editing. JM-L: data analysis. PA-A: design, construction of experimental systems, and writing—review and editing. RM-Y: conceptualization, methodology, formal analysis, investigation, writing—original draft preparation, and project administration. All authors contributed to the article and approved the submitted version.

## Funding

We appreciate the financial support granted by an internal fund from the Universidad de Guanajuato and to UNAM Project IN217322.

## Conflict of Interest

The authors declare that the research was conducted in the absence of any commercial or financial relationships that could be construed as a potential conflict of interest.

## Publisher's Note

All claims expressed in this article are solely those of the authors and do not necessarily represent those of their affiliated organizations, or those of the publisher, the editors and the reviewers. Any product that may be evaluated in this article, or claim that may be made by its manufacturer, is not guaranteed or endorsed by the publisher.

## References

[1] Mota VC Limbu P Martins CIM Eding EH Verreth JAJ. The effect of nearly closed RAS on the feed intake and growth of Nile tilapia (*Oreochromis niloticus*), African catfish (*Clarias gariepinus*) and European eel (*Anguilla anguilla*). Aquac Eng. (2015) 68:1–5. 10.1016/j.aquaeng.2015.06.002

[2] Food and Agriculture Organization (FAO). The State of World Fisheries and Aquaculture. Rome: FAO (2020).

[3] Timmons MB Ebeling JM. Recirculating Aquaculture. Cayuga Aqua Ventures. Ithaca, NY: Northeastern Regional Aquaculture Center (2010).

[4] Broom DM. Animal welfare: concepts and measurement. J Anim Sci. (1991) 69:4167–75. 10.2527/1991.69104167x 1778832

[5] Carenzi C Verga M. Animal welfare: review of the scientific concept and definition. Ital J Anim Sci. (2009) 8(sup1):21–30. 10.4081/ijas.2009.s1.21

[6] World Organization of Animal Health (OIE). Animal Welfare. (2020). Available online at: https://www.oie.int/en/what-we-do/animal-health-and-welfare/animal-welfare/ (accessed October 30, 2020).

[7] Arvizu LO Téllez ER. Animal Welfare in México: A Normative View Universidad Nacional Autónoma de México. Mexico City: UNAM (2016). p. 145.

[8] De Brlyne N. Veterinary Aspects of Aquatic Animals Health and Welfare, Aquaculture and Ornamental Fish Trade. Brussels: Federation of Veterinarians of Europe (2014).

[9] Mellor DJ Beausoleil NJ Littlewood K McLean AN McGreevy PD Jones B. The 2020 five domains model: Including human – animal interactions in assessments of animal welfare. Animals. (2020) 10:1–24. 10.3390/ani10101870 33066335 PMC7602120

[10] Noble C Gismervik K Iversen MH Kolarevic J Nilsson J Stien LH. Welfare Indicators for Farmed Atlantic Salmon: Tools for Assessing Fish Welfare. Tromso: Norweigian Seafood Research Fund (2018).

[11] Ellingsen K Grimsrud K Nielsen HM Mejdell C Olesen I Honkanen P. Who cares about fish welfare? A Norwegian study. Br Food J. (2015) 117:257–73. 10.1108/BFJ-08-2013-0223

[12] Broom DM. Cognitive ability and sentience: Which aquatic animals should be protected? Dis Aquac Org. (2007) 75:99–108. 10.3354/dao075099 17578249

[13] Damsgard B Juell JE Braastad BO. Welfare in Farmed Fish. Tromso: Norwegian Institute of Fisheries and Aquaculture Research (2006).

[14] Martins CLM Galhardo L Noble C Damsgard D Spedicato MT Zupa W. Behavioral indicators of welfare in farmed fish. Fish Physiol Biochem. (2012) 38:17–41. 10.1007/s10695-011-9518-8 21796377 PMC3276765

[15] Gutierrez-Rabadán C Spreadbury C Consuegra S García-de-Leaniz C. Development, validation and testing of an Operational Welfare Score Index for farmed lumpfish *Cyclopterus lumpus* L. Aquaculture. (2021) 531:1–12. 10.1016/j.aquaculture.2020.735777

[16] Branson EJ. 2008 Fish Welfare. Monmouthshire: Blackwell Publishing.

[17] Luo G Gao Q Wang C Liu W Sun D Li L. Growth, digestive activity, welfare, and partial cost-effectiveness of genetically improved farmed tilapia (*Oreochromis niloticus*) cultured in arecirculating aquaculture system and an indoor biofloc system. Aquaculture. (2014) 423:1–7. 10.1016/j.aquaculture.2013.11.023

[18] Nash RDM Valencia AH Jeffrey AJ. The Origin of Fulton's Condition Factor: Setting the Record Straight. Fisheries. (2006) 31:236–8.

[19] Roberts RJ. Fish Pathology. Oxford: Willey-Blackwell (2012).

[20] Volkoff H Peter RE. Feeding behavior of fish and its control. Zebrafish. (2006) 3:1–10. 10.1089/zeb.2006.3.131 18248256

[21] Stien LH Bracke MBM Folkedal O Nilsson J Oppedal F Torgersen T. Salmon Welfare Index Model (SWIM 10): a semantic model for overall welfare assessment of caged Atlantic salmon: review of the selected welfare indicators and model presentation. Rev Aquac. (2013) 5:33–57. 10.1111/j.1753-5131.2012.01083.x

[22] Food and Agriculture Organization (FAO). Aquaculture Feed and Fertilizer Resources Information System. (2019). Available online at: http://www.fao.org/fishery/affris/species-profiles/nile-tilapia/tables/en/ (accessed June 30, 2019).

[23] Sánchez JA López-Olmeda JF Blanco-Vives B Sánchez-Vázquez FJ. Effects of feeding schedule on locomotor activity rhythms and stress response in sea bream. Physiol Behav. (2009) 98:125–9. 10.1016/j.physbeh.2009.04.020 19410591

[24] Vera LM De Pedro N Gómez-Milán E Delgado MJ Sánchez-Muros MJ Madrid JA. Feeding entrainment of locomotor activity rhythms, digestive enzymes and neuroendocrine factors in goldfish. Physiol Behav. (2007) 90:518–24. 10.1016/j.physbeh.2006.10.017 17196229

[25] Gao Y Wang Z Hur JW Lee JY. Body composition and compensatory growth in Nile tilapia *Oreochromis niloticus* under different feeding intervals. Chin J Oceanol Limnol. (2015) 33:945–56. 10.1007/s00343-015-4246-z

[26] Roh HJ Park J Kim A Kim N Lee Y Kim BS. Overfeeding-induced obesity could cause potential immuno-physiological disorders in rainbow trout (*Oncorhynchus mykiss*). Animals. (2020) 10:1–15. 10.3390/ani10091499 32854279 PMC7552159

[27] Sriyasak P Chitmanat C Whangchai N Promya J Lebel L. Effect of water de-stratification on dissolved oxygen and ammonia in tilapia ponds in Northern Thailand. Int Aqua Res. (2015) 7:287–99. 10.1007/s40071-015-0113-y

[28] Katz DL Elmore JG Wild DMG Lucan SC. Jekel's Epidemiology, Bioestatistics, Preventive Medicine and Public Heatlh. Philapelphia, United States: Elseviers Saunders (2014).

[29] Thrusfield M. Veterinary Epidemiology. 4th ed. Edinburg: Wiley Blackwell (2018).

[30] Leishman EM van Staaveren N Osborne VR Wood BJ Baes CF Harlander-Matauschek A. The prevalence of integument injuries and associated risk factors among Canadian Turkeys. Front Vet Sci. (2022) 8:757776. 10.3389/fvets.2021.757776 35071378 PMC8777054

[31] Schmidt CO Kohlmann T. When to use the odds ratio or the relative risk? Int J Public Health. (2008) 53:165–7. 10.1007/s00038-008-7068-3 19127890

[32] Hisano H Barbosa PTL Hayd LA. Evaluation of Nile tilapia in monoculture and polyculture with giant fresh water prawn in biofloc technology system and in recirculation aquaculture system. Int Aqua Res. (2019) 11:335–46. 10.1007/s40071-019-00242-2

[33] Cañon-Jones HA Noble C Damsgard B Pearce GP. Investigating the influence of predictable and unpredictable feed delivery schedules upon the behaviour and welfare of Atlantic salmon parr (*Salmo salar*) using social network analysis and fin damage. Appl Anim Behav Sci. (2012) 138:132–140. 10.1016/j.applanim.2012.01.019

[34] Stejskal V Matoušek J Prokešová M Podhorec B Krištan J Policar T. Fin damage and growth parameters relative to stocking density and feeding method in intensively cultured European perch *(Perca fluviatilis L)*. J Fish Dis. (2019) 43:1–10. 10.1111/jfd.13118 31770815

[35] Pedrazzani AS Quintiliano MH Bolfe F Sans ECO Molento CFM. Tilapia on-farm welfare assessment protocol for semi-intensive production systems. Front Vet Sci. (2020) 7:1–16. 10.3389/fvets.2020.606388 33324705 PMC7723968

[36] Espinoza-Moya EA Angel-Sahagún CA Mendoza-Carrillo JM Albertos-Alpuche PJ Alvarez-González CA Martínez-Yáñez AR. Herbaceous plants as part of biological filter for aquaponic system. Aquac Res. (2016) 47:1716–26. 10.1111/are.12626

[37] García F Romera DM Gozi KS Onaka EM Fonseca FS Schalch SHC. Stocking density of Nile tilapia in cages placed in a hydroelectric reservoir. Aquaculture. (2013) 411:51–6. 10.1016/j.aquaculture.2013.06.010

[38] Coyle SD Durborow RM Tidwell JH. Anesthetics in Aquaculture. Southern Regional Aquaculture Center. Lexintong: Kentucky State University (2004).

[39] Matthews M Varga ZM. Anesthesia and Euthanasia in Zebrafish. J Inst Lab Anim Res. (2012) 53:192–204. 10.1093/ilar.53.2.192 23382350

[40] Olesen I Alfnes F Røra MB Kolstad K. Eliciting consumers'willingness to pay for organic and welfare-labelled salmon in a non-hypothetical choice experiment. Livest Sci. (2010) 127:218–26. 10.1016/j.livsci.2009.10.001

[41] Caglan A Benli K Köksal G. The acute toxicity of ammonia on tilapia (Oreochromis niloticus L) larvae and fingerlings. Turkish J Vet Anim Sci. (2003) 29:339–44. 10.3906/vet-0306-44

[42] Mengitsu SB Mulder HA Benzie JAH Komen H. A systematic literature review of the major factors causing yield gap by affecting growth, feed conversion ratio and survival in Nile tilapia (*Oreochromis niloticus*). Rev Aquac. (2020) 12:1–18. 10.1111/raq.12331

[43] Abdelghany AE Ahmad MH. Effects of feeding rates on growth and production of Nile tilapia, common carp and silver carp polycultured in fertilized ponds. Aquac Res. (2002) 33:415–23. 10.1046/j.1365-2109.2002.00689.x

[44] Dolomatov S Zukow W Dzierzanowski M Mieszkowski J Muszkieta R Klimezyk M. Roles of nitrates in the adaptation of fish to hypoxic conditions. Water Resour. (2016) 43:177–83. 10.1134/S0097807816120046

[45] Foss A Siikavuopio SI Saether BS Evensen TH. Effect of chronic ammonia exposure on growth in juvenile Atlantic cod. Aquaculture. (2004) 237:179–89. 10.1016/j.aquaculture.2004.03.013

[46] Oberle M Salomon S Ehrmaier B Richter P Lebert M Strauch SM. Diurnal stratification of oxygen in shallow aquaculture ponds in central Europe and recommendations for optimal aeration. Aquaculture. (2019) 501:482–7. 10.1016/j.aquaculture.2018.12.005

[47] Liang JY Chien YH. Effects of feeding frequency and photoperiod on water quality and crop production in a tilapia-water spinach raft aquaponics system. Int Biodeterior Biodegradation. (2013) 85:693–700. 10.1016/j.ibiod.2013.03.029

[48] Castillo JDA do Nascimento TMT Mansano CFM Sakomura NK da Silva NP Fernandes JDK. Determinig the daily digestive protein intake for Nile tilapia at different growth stages. Boletim do Instituto de Pesca. (2017) 44:54–66. 10.20950/1678-2305.2017.54.63

[49] Van Trung D Diu NT Hao NT Glencross B. Development of a nutritional model to define the energy and protein requirements of tilapia, *Oreochromis niloticus*. Aquaculture. (2011) 320:69–75. 10.1016/j.aquaculture.2011.07.029

[50] Houlijan D Boujard T Jobling M. Food Fish Intake. Oxford: Blackwell Science (2001).

[51] Saravan S Geurden I Figueiredo-Silva AC Kaushik SJ Haidar MN Verreth JAJ. Control of voluntary feed intake in fish: a role for dietary oxygen demand in Nile tilapia (*Oreochromis niloticus*) fed diets with different macronutrient profiles. Br J Nutr. (2012) 108:1519–29. 10.1017/S0007114511006842 22221412

[52] El-Dakar AY Shlaby SM Mostafa EE Abdel-Aziz MF. The optimum level of dietary protein and feeding for improving the growth performance and feed efficiency of juveniles Hybrid tilapia (*Oreochromis niloticus* × *Oreochromis aurea*) reared in brackish water. Egyp J Aquac Biol Fish. (2021) 25:839–56. 10.21608/ejabf.2021.195285

[53] Gonçalves-de-Freitas G Bolognesi MC dos Santos Gauy AC Brandão ML Giaquinto PC Fernandes-Castilho M. Social Behavior and Welfare in Nile Tilapia. Fishes. (2019) 4:2–14. 10.3390/fishes4020023

[54] Almazán-Rueda P Schrama JW Verreth JAJ. Behavioural responses under different methods and light regimes of the African catfish (Clarias gariepinus) juveniles. Aquaculture. (2004) 231:349–57. 10.1016/j.aquaculture.2003.11.016

[55] Hoyle I. Oidmann B, Ellis T, Turnbul J, North B, Nikolaidis J, Nowles TG. A validated macroscopic key to assess fin damage in farmed rainbow trout (*Oncorhynchus mykiss*). Aquaculture. (2007) 270:142–8. 10.1016/j.aquaculture.2007.03.037

[56] Tesfahun A Tesmegen M. Food and feeding habits of Nile tilapia *Oreochromis niloticus* (L) in Ethiopian water bodies: a review. Int J Fish Aquat Stud. (2018) 6:43–7. Available online at: https://www.fisheriesjournal.com/archives/2018/vol6issue1/PartA/5-6-54-506.pdf

[57] Rey S Tresaurer J Pattillo C McAdam BJ. Using model selection to choose a size-based condition indexthat is consistent with operational welfare indicators. J Fish Biol. (2021) 99:782–95. 10.1111/jfb.14761 33890676

[58] Bemis WE Hilton EJ Brown B Arrindeli R Richmond AR Little CD. Methods for Preparing Dry, Partially Articulated Skeletons of Osteichthyans, with Notes on Making Ridewood Dissections of the Cranial Skeleton. Copeia. (2004) 3:603–9. 10.1643/CI-03-054R1

[59] North BP Turnbull JF Ellis T Porter MJ Migaud H Bron J. The impact of stocking density on the welfare of rainbow trout (*Onchorynchus mykiss*). Aquaculture. (2006) 255:466–79. 10.1016/j.aquaculture.2006.01.004

[60] Turnbull J Bell A Adams C Bron J Huntingford F. Stocking density and welfare of cage farmed Atlantic salmon: application of a multivariate analysis. Aquaculture. (2005) 243:121–32. 10.1016/j.aquaculture.2004.09.022

[61] Pelis RM McCormick SD. Fin development in stream- and hatchery-reared Atlantic salmon. Aquaculture. (2003) 220:525–36. 10.1016/S0044-8486(02)00625-7

[62] St.Hilaire S Ellis T Cooke A North BP Turnbull JF Knowles T. Fin erosion on rainbow trout on commercial trout farms in the United Kingdom. Vet Rec. (2006) 159:446–51. 10.1136/vr.159.14.446 17012609

[63] Latremouille DN. Fin erosion in aquaculture and natural environments. Rev Fish Sci. (2003) 11:315–35. 10.1080/1064126039025574525469952

[64] Van de Nieuwegiessen PG Boerlange AS Verreth JAJ Schrama JW. Assessing the effects of a chronic stressor, stocking density, on welfare indicators of juvenile African catfish, *Clarias gariepinus* Burchell. Appl Anim Behav Sci. (2008) 115:233–43. 10.1016/j.applanim.2008.05.008

[65] Noble C Kadri S Mitchell DF Huntingford FA. Growth, production and fin damage in cage-held 0 + Atlantic salmon pre-smolts (Salmo salar L) fed either a) on demand, or b) to a fixed satiationrestriction regime: data from a commercial farm. Aquaculture. (2008) 275:163–8. 10.1016/j.aquaculture.2007.12.028

[66] Noble C Kadri S Mitchell DF Huntingford FA. The effect of feed regime on the growth and behaviour of 1 + Atlantic salmon postsmolts (*Salmo salar* L.) in semi-commercial sea cages. Aquac Res. (2007) 38:1686–91. 10.1111/j.1365-2109.2007.01833.x

[67] Noble C Mizusawa K Suzuki K Tabata M. The effect of differing self-feeding regimes on the growth, behaviour and fin damage of rainbow trout held in groups. Aquaculture. (2007) 264:214–22. 10.1016/j.aquaculture.2006.12.028

[68] Person-Le Ruyet J Le Bayo N Gros S. How to assess fin damage in rainbow trout, *Onchorynchus mykiss*? Aquat Living Resour. (2007) 20:191–5. 10.1051/alr:2007031

